# Quantifying differences in plant architectural development between hybrid potato (*Solanum tuberosum*) plants grown from two types of propagules

**DOI:** 10.1093/aob/mcad194

**Published:** 2023-12-14

**Authors:** Jiahui Gu, Paul C Struik, Jochem B Evers, Narawitch Lertngim, Ruokai Lin, Steven M Driever

**Affiliations:** Centre for Crop Systems Analysis, Wageningen University & Research, PO Box 430, 6700 AK Wageningen, The Netherlands; Centre for Crop Systems Analysis, Wageningen University & Research, PO Box 430, 6700 AK Wageningen, The Netherlands; Centre for Crop Systems Analysis, Wageningen University & Research, PO Box 430, 6700 AK Wageningen, The Netherlands; Centre for Crop Systems Analysis, Wageningen University & Research, PO Box 430, 6700 AK Wageningen, The Netherlands; Centre for Crop Systems Analysis, Wageningen University & Research, PO Box 430, 6700 AK Wageningen, The Netherlands; Centre for Crop Systems Analysis, Wageningen University & Research, PO Box 430, 6700 AK Wageningen, The Netherlands

**Keywords:** Plant architecture, propagule type, branching, potato growth and development, biomass allocation, true potato seed, seedling tuber, *Solanum tuberosum*

## Abstract

**Background and Aims:**

Plants can propagate generatively and vegetatively. The type of propagation and the resulting propagule can influence the growth of the plants, such as plant architectural development and pattern of biomass allocation. Potato is a species that can reproduce through both types of propagation: through true botanical seeds and seed tubers. The consequences of propagule type on the plant architectural development and biomass partitioning in potatoes are not well known. We quantified architectural differences between plants grown from these two types of propagules from the same genotype, explicitly analysing branching dynamics above and below ground, and related these differences to biomass allocation patterns.

**Methods:**

A greenhouse experiment was conducted, using potato plants of the same genotype but grown from two types of propagules: true seeds and seed tubers from a plant grown from true seed (seedling tuber). Architectural traits and biomass allocation to different organs were quantified at four developmental stages. Differences between true-seed-grown and seedling-tuber-grown plants were compared at the whole-plant level and at the level of individual stems and branches, including their number, size and location on the plant.

**Key Results:**

A more branched and compact architecture was produced in true-seed-grown plants compared with seedling-tuber-grown plants. The architectural differences between plants grown from true seeds and seedling tubers appeared gradually and were attributed mainly to the divergent temporal–spatial distribution of lateral branches above and below ground on the main axis. The continual production of branches in true-seed-grown plants indicated their indeterminate growth habit, which was also reflected in a slower shift of biomass allocation from above- to below-ground branches, whereas the opposite trend was found in seedling-tuber-grown plants.

**Conclusions:**

In true-seed-grown plants, lateral branching was stronger and determined whole-plant architecture and plant function with regard to light interception and biomass production, compared with seedling-tuber-grown plants. This different role of branching indicates that a difference in preference between clonal and sexual reproduction might exist. The divergent branching behaviours in true-seed-grown and seedling-tuber-grown plants might be regulated by the different intensity of apical dominance, which suggests that the control of branching can depend on the propagule type.

## INTRODUCTION

Many plants can reproduce either sexually or clonally. Potato (*Solanum tuberosum*) is a species that can naturally reproduce via both reproductive modes. Potato plants can reproduce vegetatively from tubers that are modified stem organs. In contrast, the true potato seeds (TPS), which are botanical seeds generated via sexual reproduction, can also form tuber-producing plants. These true seeds, however, were rarely used in commercial production in the past, owing to their large genetic variation and poor vigour (Almekinders *et al.*, 1996, 2009). Recently, diploid hybrid breeding revived the use of TPS for potato propagation (Lindhout *et al.*, 2011, 2018). Genetically uniform and more vigorous cultivars can now be developed based on true seed, meaning that the progress in breeding has been substantially accelerated (Eggers *et al.*, 2021; Struik *et al.*, 2023). The disease-free phytosanitary status, the low costs for storage and transportation, and the high multiplication rate make TPS an attractive alternative to seed tubers in potato production (Simmonds, 1997; Bradshaw, 2022).

The TPS technology can result in different types of starting materials to produce seed tubers or ware potatoes: true seeds, transplants (i.e. TPS seedlings), seedling tubers (tubers derived from true-seed-grown plants) and seed tubers (tubers produced from seedling-tuber-grown plants) (Struik and Wiersema, 1999; Stockem *et al.*, 2020; Kacheyo *et al.*, 2021). In potato, seedling tubers are the tubers produced directly from true-seed-grown plants, which can produce the next generation clonal plants, whereas plants grown from seed tubers are the new generation of seedling-tuber-grown plants. Various types of propagules, however, produce plants that differ in growth and development. For example, architectural differences were observed between true-seed-grown and seedling-tuber-grown plants, including differences in the number of main stems and types of branches, even when plants were genetically identical and grown in the same conditions (Kacheyo *et al.*, 2021). These differences greatly influenced biomass production, causing the field-grown crops from true seed to produce lower yields than crops from seedling tubers of the same genotype (de Vries *et al.*, 2023). Such an effect of propagule type in crops from the same genotype has also been reported in other species, such as some horticultural crops and fruit trees (i.e. Rodrigues *et al.*, 2007; Albrecht *et al.*, 2017). Lower tuber yield was produced by botanical-seed-propagated Jerusalem artichoke (*Helianthus tuberosus*) owing to the delayed canopy closure compared with seed-tuber-grown plants (Rodrigues *et al.*, 2007). Root architectural traits, including the number of lateral roots and specific root length, developed differently among young citrus trees propagated from true seed, stem cuttings or tissue culture (Albrecht *et al.*, 2017). These studies suggest that vegetative propagules influence biomass production and key architectural traits differently compared with true seeds.

The architecture of a plant is a crucial determinant of resource acquisition and influences plant growth and biomass partitioning. Plant architecture is genotype specific, and it can be modified by endogenous processes and environmental conditions (Reinhardt and Kuhlemeier, 2002; Barthélémy and Caraglio, 2007; Pierik *et al.*, 2021). Hence, investigating plant architectural development is a premise to gain more insights into the mechanisms that cause differences in contrasting types of propagules. Potato is a good species to be studied for this purpose, especially because of the success of the novel hybrid TPS technology, providing different types of propagules from the same genotype more easily than in other plant species.

As in other plant species, the architecture of potato plants is determined mainly by the number and position of axillary meristems, control of bud break and the subsequent dynamics in branch growth (Teichmann and Muhr, 2015). The potato plant forms a hierarchical, branched system in both true-seed-grown and seedling-tuber-grown plants. Above ground, green leaves, stems and lateral branches produce photoassimilates to maintain their own growth and/or produce new organs. Below ground, lateral branches, including the horizontally growing stolons and tubers (swollen stolons), compete with above-ground branches for assimilates and store biomass. The dynamic interactions between above- and below-ground branches influence the growth and development of the whole plant (Vos, 1999). Therefore, to describe how the architectural differences develop in the two contrasting propagules, the quantification of stem branching dynamics is crucial.

To date, branching dynamics in potato, especially for true-seed-grown plants, have not been quantified. In plants grown from tubers (including seedling tubers and seed tubers), the shoot stem development is quantified as a function of the rates and durations of initiation of leaf and flower primordia (Almekinders and Struik, 1996). The below-ground branch development can be described as changes in the number of stolons and tubers over time and as the delays between stolon and tuber initiation (Vreugdenhil and Struik, 1989; Struik, 2007). However, how the interactions between above- and below-ground branching affect whole-plant architecture and biomass partitioning and whether such relationships also apply for true-seed-grown plants, is unclear. Moreover, the architectural differences between tuber-grown and true-seed-grown plants have been described only qualitatively: tuber-grown plants normally produce more than one main stem and develop below-ground basal branches that can bear tubers, whereas true-seed-grown plants have only one main axis, and no below-ground basal branches are produced (Vos, 1995; Struik and Wiersema, 1999; Struik, 2007; Kacheyo *et al.*, 2021).

Here, we compared the architectural development between potato plants grown from true seeds and seedling tubers from the same genotype. This analysis was conducted for greenhouse-raised plants from both types of propagules, which is an approach able to identify the spatial distribution of plant organs and the relationships between them (Barthélémy and Caraglio, 2007). Using this method, we aimed: (1) to identify and quantify differences between true-seed-grown and seedling-tuber-grown plants in branching patterns above and below ground; and (2) to relate these differences to biomass allocation patterns.

## MATERIALS AND METHODS

### Plant materials

Plant material was provided by the Dutch potato breeding company Solynta (Wageningen, the Netherlands). True seeds and seedling tubers of the same genotype, SOLHY007, were used, which were obtained from the 2021 growing season. Young plants grown from true seeds and seedling tubers were raised separately before the experiment to ensure uniformity of plant material at the start of the experiment. True seeds were sown in plug trays with one seed per plug in the greenhouse nursery on 24 June 2022. Each plug (28 mm diameter × 32 mm height) contained a mixture of substrates, with 30 % white peat, 50 % coco peat and 20 % perlite. Seedling tubers (35–45 mm square size) were taken out from cool storage at 4 °C on the same day and started presprouting, as described by Struik and Wiersema (1999). Seventeen days later, plants grown from true seeds with four or five true leaves and seedling tubers with two green sprouts per tuber of homogeneous size were selected, respectively, for transplanting. Ten-litre pots (26.5 cm in diameter and 21 cm in height) were used, filled with a mixture of black soil and potting soil (1:1) up to 2 cm below the rim of the pot. On 11 July 2022, individual true-seed-grown plants and seedling tubers were transplanted to each pot at 5 cm depth from the top of the soil.

### Experimental design

A pot experiment was conducted in an environmentally controlled greenhouse at Wageningen University and Research, Wageningen, the Netherlands (52°N, 6°E). A randomized complete block design was implemented, with the treatment of two propagule types and four replicates (blocks) positioned against the light gradient. Each replicate contained ten pots for each propagule type, which comprised a total of 80 pots. Within each replicate, pots with plants were randomly placed on moveable tables with equal distance between pots to prevent shading from neighbouring plants. After the emergence of seedling-tuber-grown plants (~1 week after transplanting), only one main stem was maintained per plant, and extra ones were removed to make them comparable with single-stemmed true-seed-grown plants. The experiment lasted until 12 weeks after transplanting.

The environment in the greenhouse was set with a 16 h photoperiod, an average temperature of 20 °C during the day and 15 °C at night, and a relative humidity of 65 %. Natural light was used during the photophase, and additional lighting by high-pressure sodium lamps was given when outdoor solar radiation was <400 W m^−2^ and switched off when it was >500 W m^−2^. Plants were supplied with 400 mL nutrient solution (EC [Electrical Conductivity] = 4.4 dS m^−1^; pH = 6.5) once a week for the first 4 weeks and twice a week for the following 4 weeks. The nutrient solution contained, per litre, 2.4 g N, 0.6 g P, 3.5 g K, 0.46 g Ca, 0.28 g Mg and trace elements (2.0 g MnSO_4_.H_2_O, 3.0 g H_3_BO_3_, 0.5 g ZnSO_4_.7H_2_O, 0.1 g CuSO_4_.5H_2_O and 0.1 g Na_2_MoO_4_.2H_2_O).

### Plant architectural traits and biomass measurements

Destructive measurements were taken every 3 weeks (weeks 3, 6, 9 and 12 after transplanting). Each time, two plants per replicate (eight plants in total) were harvested for each of the two propagule types. Architectural traits were determined on individual plants, including plant height, main stem height, the number of nodes and leaves on the main stem and individual leaf area, the number of branches above ground, the length, number of leaves and leaf area of individual branches, the number of stolons and tubers (branches below ground) and the individual tuber size and fresh weight.

**Fig. 1.**
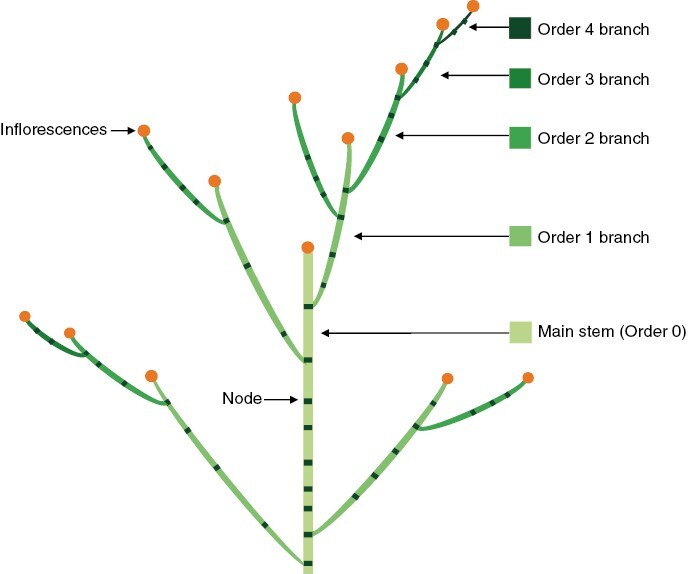
Illustration of stems and branches assigned with specific branching orders. Adapted from Vos (1995).

Plant height was determined from the top leaf to soil level. The main-stem height was measured from the top node to soil level. The number of branches above ground was counted at every node position along the main stem and separated according to their branching order. Adapted from Vos (1995), the main stem that grew from a true seed or a seedling tuber was assigned as order 0 axis, and the branches developed from leaf axils on the main stem were order 1 axes; order 2 branches were born below the inflorescence of the order 1 branches (Fig. 1). The same rules applied for higher-order branches. In this study, the maximum branching order was limited to order 4. Branches were recorded when >5 cm in length. The length of a branch was measured from its tip to its base that connected it with its parent stem. On each individual branch, the number of fully expanded leaves (>1 cm in length) was recorded, and the total leaf area was measured. The leaf area of the leaves on the main stems was determined individually based on their node positions. The stem length was determined using a ruler. Leaf area was measured using a LI-COR-3100 (LI-COR BioScience, Lincoln, NE, USA).

Below-ground branches were grouped into stolons per plant and tubers per plant, because there was no clear pattern found in the development of stolons or tubers (Struik, 2007). Tubers with a diameter twice that of their extending stolons were recorded. The size of individual tubers was assessed based on their transverse diameter, using square meshes with edge lengths of 25, 35, 45, 55 and 65 mm, respectively. Tubers (with short end) unable to pass through a specific mesh were categorized into the size class that had a lower limit corresponding to this mesh size and an upper limit corresponding to the next larger mesh size. Individual tuber fresh weight was determined using an analytical balance.

After that, the leaves and stems from each branching order, berries, stolons, roots and tubers were collected separately per plant and oven dried at 105 °C to a constant weight. The biomass of those plant parts was determined using an electric balance.

### Statistical analyses

Given that independent plants were harvested destructively at the four developmental stages, a two-way ANOVA for a randomized complete block design was performed to test the differences in representative plant traits between treatment and over time. The effect of propagule type and measurement week and their interaction were assessed. To identify the differences between propagule types, for each variable, data were grouped by measurement week, and a one-way ANOVA for a randomized complete block design of simple main effect of propagule type was done with statistical significance of *P* < 0.05. The pooled error sum of squares and degree of freedom were used to specify the ANOVA model. The correlations among variables were analysed by a Pearson test for each propagule type, to test relationships between above-ground branching and below-ground branching traits. A principal component analysis was conducted for those variables to summarize the traits contributing the most to whole-plant architecture for each propagule type. All analyses were performed using R (v.4.1.2; R Core Team, 2021).

## RESULTS

### Whole-plant architecture

**Fig. 2.**
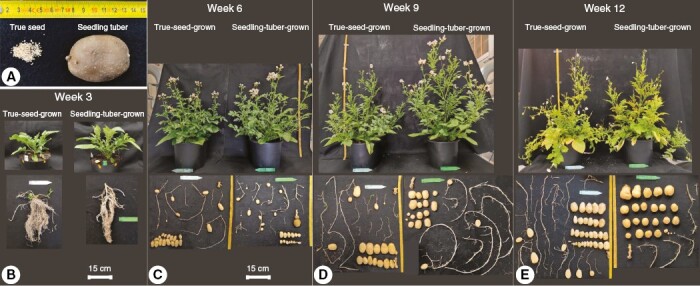
Photographs of true seeds and a seedling tuber (A) and true-seed-grown and seedling-tuber-grown plants at four developmental stages (B–E). One representative plant for each propagule type out of eight plants was selected at each stage. The above-ground part includes leafy branches from different branching orders, whereas the below-ground part contains mainly stolons and tubers.

An overview of whole-plant architectures is illustrated for plants grown from true seeds and seedling tubers, respectively, at four developmental stages (3, 6, 9 and 12 weeks after transplanting; Fig. 2). True-seed-grown and seedling-tuber-grown plants produced very similar architectures at an early stage (i.e. week 3; Fig. 2B), and their distinct architectures started to appear at week 6 (Fig. 2C–E).

In week 3, plants grown from two propagule types arrived at growth stages 204–205 (BBCH [Biologische Bundesanstalt, Bundessortenamt and CHemical Industry] scale; Kacheyo *et al.*, 2021). Above ground, the leaf development on the main stem was dominant. At the basal part of the main stem, four to five lateral branches were visible. Below ground, several stolons grew horizontally and started to be swollen at the tips, which initiated the growth of tubers. By week 9, true-seed-grown plants had grown to the maximum height, but because of the earlier senescence of the main stem, plants sagged in week 12. In contrast, seedling-tuber-grown plants were still stretching until the end. When the growth of the main stem was almost halted, the development of branches was enhanced, in terms of the number and size of branches. Above-ground branches developed much faster in seedling-tuber-grown plants up to week 9, compared with true-seed-grown plants, whereas the gap narrowed and disappeared in week 12.

Below the soil, the development of stolons and tubers happened simultaneously. The number of stolons was fixed at the beginning of branch development (before week 3), with slightly more stolons in true-seed-grown plants than in seedling-tuber-grown plants. Consequently, more tubers were produced at final harvest in true-seed-grown plants compared with seedling-tuber-grown plants.

Overall, at the whole-plant level only a few differences in architectural traits were found between the plants grown from two propagule types, when considering the sum of those variables per plant (Table 1). The significant week effect on almost all the listed traits simply described the growth of plants over time. The architectural differences between the two propagule types existed only in the traits plant height, main stem height, the number of stolons and the number of tubers. In other traits, the differences emerged gradually as plants grew older, but were not statistically significant between the two propagule types (Supplementary Data Table S1). This led our investigation focus on lateral branches.

**Table 1.** ANOVA for main effects of propagule type and week and their interaction for whole-plant architectural traits per plant (mean values of eight plants).

Plant traits	Propagule type effect	Week effect	Propagule type × week
Plant height (cm)	0.005**	<0.001***	0.031*
Main stem height (cm)	<0.001***	<0.001***	<0.001***
Number of branches above-ground	0.350	<0.001***	0.088
Number of leaves	0.588	<0.001***	0.224
Green leaf area (cm^2^)	0.257	<0.001***	0.250
Number of stolons	0.037*	0.273	0.646
Number of tubers	0.002**	<0.001***	0.110
Total number of branches (above-ground + below-ground branches)	0.125	<0.001***	0.542
Total branch length (cm)	0.286	<0.001***	0.090

Statistical significances are indicated by asterisks (*P < 0.05, **P < 0.01, ***P < 0.001).

### Temporal–spatial distribution of branches

#### Above-ground stems and branches.

 The temporal distribution of stems or branches can be described from the perspective of branching order. The production of branches is attributable to the outbreak of axillary buds on the stem nodes. The number of branches from higher branching order is determined by the number of nodes on their parent stem or branches. The main stem (order 0 axis) therefore provides the basis of the development of all branches. True-seed-grown plants produced significantly fewer nodes on the main stem than seedling-tuber-grown plants (Table 2). This implies that fewer order 1 branches could potentially be produced in true-seed-grown plants, and thereafter higher-order branches; however, the number of branches on every branching order was similar to that found in seedling-tuber-grown plants (Supplementary Data Table S2). Comparing across branching orders, order 2 branches contributed the most to the total number of branches for both propagule types, which, however, had a significantly higher quantity in true-seed-grown plants. In total, a marginally higher number of branches developed in true-seed-grown plants than in seedling-tuber-grown plants.

**Table 2.** Above-ground architectural traits per plant (mean **± **s.d.) between true-seed-grown and seedling-tuber-grown plants (*n* = 8). Data were collected in week 12, as development of branches above ground reached their maximum.

Plant traits	True-seed-grown plants	Seedling-tuber-grown plants
Number of nodes on main stem	**15.9 ± 1.0** ^**a**^	**17.2 ± 1.6** ^**b**^
Average internode length on main stem (cm)	**1.6 ± 0.4** ^**a**^	**2.3 ± 0.6** ^**b**^
Number of branches above ground	38.0 ± 11.1	34.9 ± 13.2
Number of branches/number of main stem nodes ratio	2.4 ± 0.7	2.0 ± 0.7
**Based on branching order** ^1^		
Order 1 branches	10.4 ± 1.1	9.9 ± 0.6
Order 2 branches	**16.5 ± 4.7** ^**b**^	**12.6 ± 3.0** ^**a**^
Order 3 branches	9.3 ± 5.8	10.1 ± 7.7
Order 4 branches	1.9 ± 1.7	2.3 ± 2.8
**Based on location on main stem** ^2^		
*Basal branches*	24.0 ± 7.7	25.5 ± 9.8
*Apical branches*	**14.0 ± 4.6** ^**b**^	**9.4 ± 3.7** ^**a**^

Different letters indicate statistical significances (values in bold) between the two propagule types.

^1^Distribution of branches based on branching order.

^2^Distribution of branches based on their locations on the main stem.

**Fig. 3.**
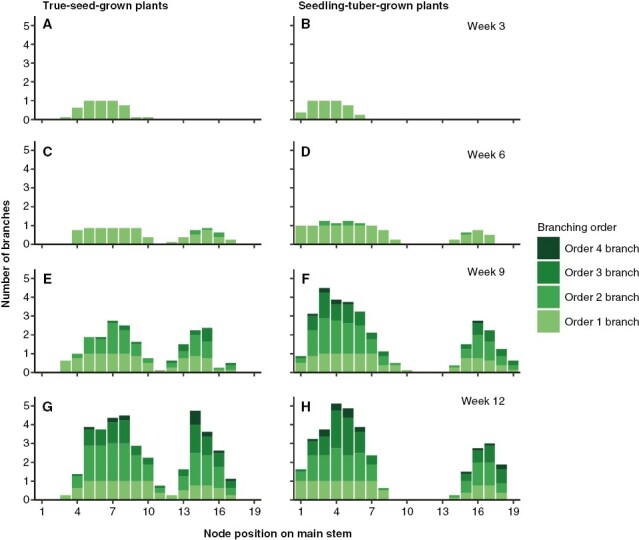
Average number of branches from different branching orders per main stem node in true-seed-grown plants and seedling-tuber-grown plants (*n* = 8) at four developmental stages. Node position 1 is at the bottom of the main stem.

The spatial distribution of branches was depicted based on their locations on the main stem, on which fewer branches were produced at the apical part in seedling-tuber-grown plants (Table 2). More specifically, the number of branches from different orders along the main axis (from bottom to top) is shown in Fig. 3. The development of order 1 branches started from basal nodes, and they grew acropetally, from node 1 to 10 (Fig. 3A, B). After the main stem terminated into inflorescence, more order 1 branches developed from top nodes and grew basipetally (Fig. 3C, D). Therefore, a gap between two peaks can be seen in the distribution of branches. Later, the higher-order branches developed from the nodes below the inflorescences of their parent branches, which showed the sympodial branching pattern (Fig. 3E–H). This is the common pattern of branch development shared between true-seed-grown plants and seedling-tuber-grown plants.

Nevertheless, branches were distributed differently along the main stem between plants grown from the two propagule types. First, although the quantity of branches was the same, branches were located closer to each other along the main stem in true-seed-grown plants, because of the shorter internode length on the main stem (Table 2). Second, seedling-tuber-grown plants started to produce higher-order sympodial branches earlier, and the duration of the branch production was also shorter; the number of branches corresponding to each branching order was attained rapidly once the development of that specific branching order was initiated (Fig. 3E, F, H). In contrast, in true-seed-grown plants, the production of new branches was continuous, without apparent signs of cessation, especially in the order 2 branches. They also produced two or more order 2 branches from each order 1 branch, which spread among most node positions (Fig. 3E, G). Lastly, there were many more apical branches developed from true-seed-grown plants, which mainly comprised higher-order branches, whereas seedling-tuber-grown plants allocated a large proportion of branches to the basal nodes (Fig. 3G, H).

**Fig. 4.**
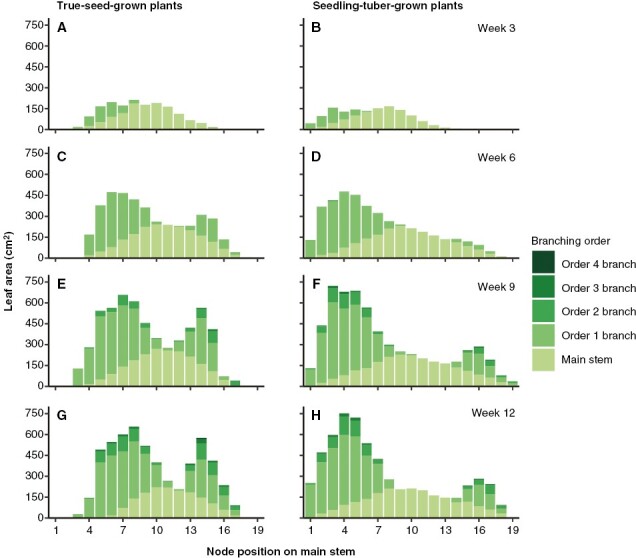
Average leaf area (in centimetres squared) distributed on different branching orders per node position on the main stem in true-seed-grown plants and seedling-tuber-grown plants (*n* = 8). Comparisons are made for four developmental stages. Node position 1 is at the bottom of the main stem.

Likewise, the distribution of leaf area along the main axis also followed the branch number distribution (Fig. 4). From the aspect of branching order, for both propagule types, main stem leaves contributed most of the whole plant leaf area until week 6, accounting for >50 % of total leaf area, which was overtaken by order 1 branch leaves at later stages (Supplementary Data Table S1). The only difference in leaf area from the main stem was attributable to the earlier senescence of main stem leaves in true-seed-grown plants, whereas on order 2, 3 and 4 branches, although not statistically significant, relatively more leaf area was produced.

In week 12, along the main stem, branch leaf area was distributed more evenly on the basal part in true-seed-grown plants, and a decreasing gradient on the apical part was observed (Fig. 4G). In seedling-tuber-grown plants, the distribution of basal leaf area displayed a bell shape and accounted for about two-thirds of the total leaf area, whereas apical leaves made little contribution to the total, and the differences in leaf area among each node position were also larger (Fig. 4H).

#### Below-ground branches.

Large differences in the number and size of below-ground branches (stolons and tubers) between the two propagule types were observed ~9 weeks after transplanting. Thereafter, the number of stolons and tubers did not change, whereas tuber bulking continued. A slightly higher number of stolons and more tubers produced per stolon resulted in a significantly higher number of tubers in true-seed-grown plants (Table 3). Total tuber fresh weight was higher in true-seed-grown plants in week 9, but thereafter tuber bulking was enhanced to a larger extent in seedling-tuber-grown plants. On average, individual tuber fresh weight was lower in true-seed-grown plants (28.1 g) than in seedling-tuber-grown plants (40.3 g) at final harvest.

**Table 3.** Number of stolons and tubers per plant (mean **± **s.d.) for true-seed-grown and seedling-tuber-grown plants (*n* = 8).

Plant traits	Week 9		Week 12	
	True-seed-grown plants	Seedling-tuber-grown plants	True-seed-grown plants	Seedling-tuber-grown plants
Number of stolons	13.1 ± 3.2	11.2 ± 1.5	11.6 ± 2.6	10.5 ± 1.8
Number of tubers	**31.1 ± 10.2** ^**b**^	**19.8 ± 8.5** ^**a**^	**31.4 ± 8.0** ^**b**^	**24.6 ± 6.3** ^**a**^
Ratio of tuber number to stolon number	2.4 ± 0.5	1.8 ± 0.7	2.8 ± 1.0	2.4 ± 0.7
Total tuber fresh weight (g)	**596 ± 67.8** ^**b**^	**496 ± 128** ^**a**^	**830 ± 103** ^**a**^	**927 ± 122** ^**b**^
Average tuber fresh weight (g)	20.5	27.9	**28.1** ^**a**^	**40.3** ^**b**^

Different letters indicate statistical significances between the two propagule types for week 9 and week 12, respectively. Statistically different values are in bold.

**Fig. 5.**
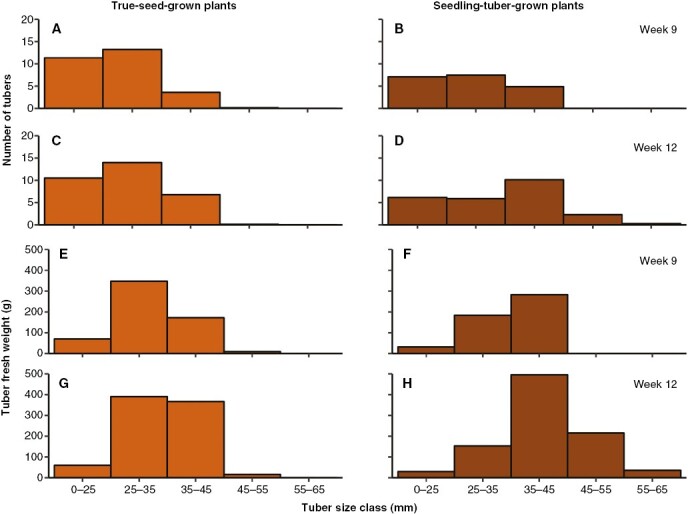
The distribution of the number of tubers (A–D) and tuber fresh weight (in grams; E–H) in five tuber size classes in weeks 9 and 12, for true-seed-grown and seedling-tuber-grown plants (mean values of eight plants).

However, given that tuber production is a continuous process, some tubers might be produced earlier or later than others, and individual tubers vary greatly in size. Therefore, the number of tubers and their individual fresh weight are normally categorized in several size classes to describe their distribution (Fig. 5). Tuber size is normally measured by the square size, which is the transversal diameter of the tuber short end. Tubers with square size <25 mm are usually too small to contribute to total tuber yield and therefore grouped together. Tubers larger than that were categorized into size classes with 10 mm increments.

In true-seed-grown plants, the total number of tubers and their distribution among each size class were fixed at week 9 (Fig. 5A, C; Supplementary Data Fig. S1), when most tubers had a small size between 0–25 and 25–35 mm. In contrast, tuber fresh weight was still increasing, especially in size classes between 25–35 and 35–45 mm, which accounted for a large proportion of the total tuber fresh weight (Fig. 5E, G). Although the total number of tubers also stopped increasing in seedling-tuber-grown plants, their distribution shifted to a larger size (Fig. 5B, D); ~20 % of the total number of tubers bulked to the larger size classes of 35–45 and 45–55 mm. Meanwhile, the total tuber fresh weight almost doubled, in which the tuber size of 35–45 mm contributed the largest proportion (Fig. 5F, H).

At the whole-plant level, the same total number of branches and total leaf area per plant were produced in true-seed-grown and seedling-tuber-grown plants. However, the distribution of these leafy branches over time and space was distinctly different between the two propagule types. Collectively, a higher contribution of branches to total stem length and total leaf area was found in true-seed-grown plants (Supplementary Data Table S1). Consequently, a more compact and more branched above-ground architecture was produced in true-seed-grown plants than in seedling-tuber-grown plants.

### Biomass allocation in different branches and other organs

The number and size distribution of branches influenced biomass production and partitioning, and vice versa. Although the total biomass per plant was not significantly different between plants grown from the two propagule types, the biomass was allocated differently into different plant parts (Table 4). As plants grew, biomass accumulated over time in each organ, which was shown in the significant week effect. The strong interactions between propagule type and week in most plant parts indicated that the biomass allocation pattern in the two types of plants changed through different growth stages.

**Table 4.** ANOVA for main effects of propagule type and week and their interaction for biomass partitioned to different parts per plant (in grams; mean values of eight plants).

Plant traits	Propagule type effect	Week effect	Propagule type × week
**Total plant biomass (g)**	0.481	<0.001***	0.217
**Above-ground part (g)**	0.105	<0.001***	<0.001***
Order 0 (main stem)^a^	<0.001***	<0.001***	<0.001***
Order 1 branches^a^	0.093	<0.001***	<0.002**
Order 2 branches^a^	0.987	<0.001***	0.004*
Order 3 branches^a^	0.523	<0.001***	0.023*
Order 4 branches^a^	0.970	<0.001***	0.255
Berries	0.027*	<0.001***	0.058
**Below-ground part (g)**	0.201	<0.001***	0.003**
Stolons	0.321	<0.001***	0.599
Roots	<0.001***	<0.001***	0.030*
Tubers	0.112	<0.001***	0.009**

Statistical significances are illustrated by asterisks (*P < 0.05, **P < 0.01, ***P < 0.001).

^a^Including all green leaves and stems belonging to this branching order.

**Fig. 6.**
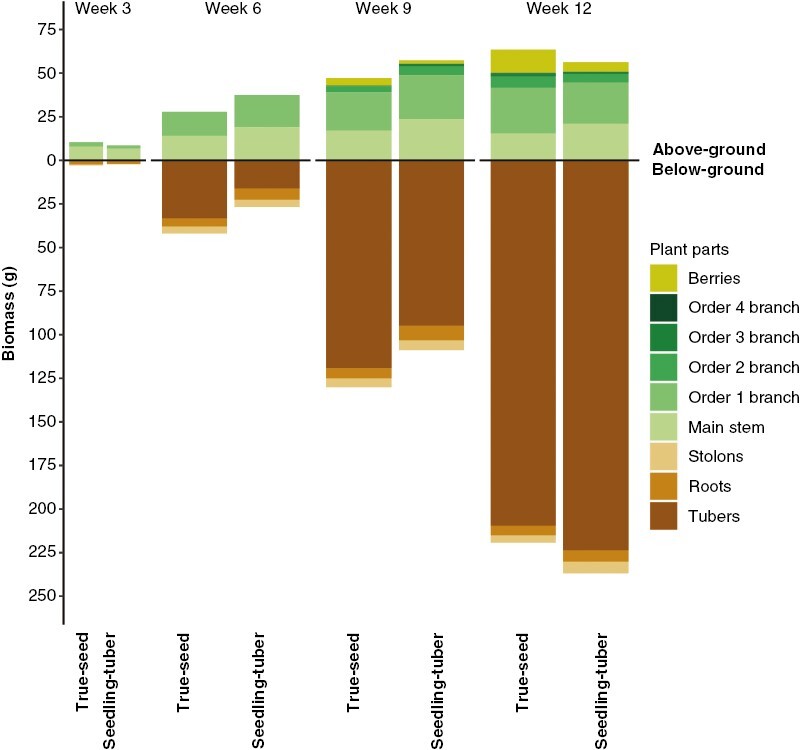
Biomass (in grams) partitioned to different parts per plant at four developmental stages for true-seed-grown and seedling-tuber-grown plants (mean values of eight plants). Biomass allocation was separated as the above-ground (upper panel) and below-ground part (lower panel).

Therefore, the biomass allocation to different organs was compared between the two propagule types for each harvest week (Fig. 6; Supplementary Data Table S3). In line with the architectural differences between true-seed-grown and seedling-tuber-grown plants, the pattern of biomass partitioning started to diverge at week 6. True-seed-grown plants invested more biomass into below-ground and less to above-ground parts until week 9, compared with seedling-tuber-grown plants. This was attributable to more biomass accumulated in tubers and less in the main stem and order 1 branches, which are the three parts contributing the largest proportions of plant biomass below and above ground, respectively.

From week 9 to 12, an opposite trend emerged. Above-ground biomass was higher in true-seed-grown plants, which contributed mainly to the larger number of berries, the true-seed-bearing organs after flowering at the tip of stems and branches. Fewer berries were produced in seedling-tuber-grown plants, and more biomass was transported to below-ground organs (stolons, roots and tubers), although compared with true-seed-grown plants, no statistical differences were found in each of these organs separately (Supplementary Data Table S3).

### Associations between branches above ground and below ground

**Fig. 7.**
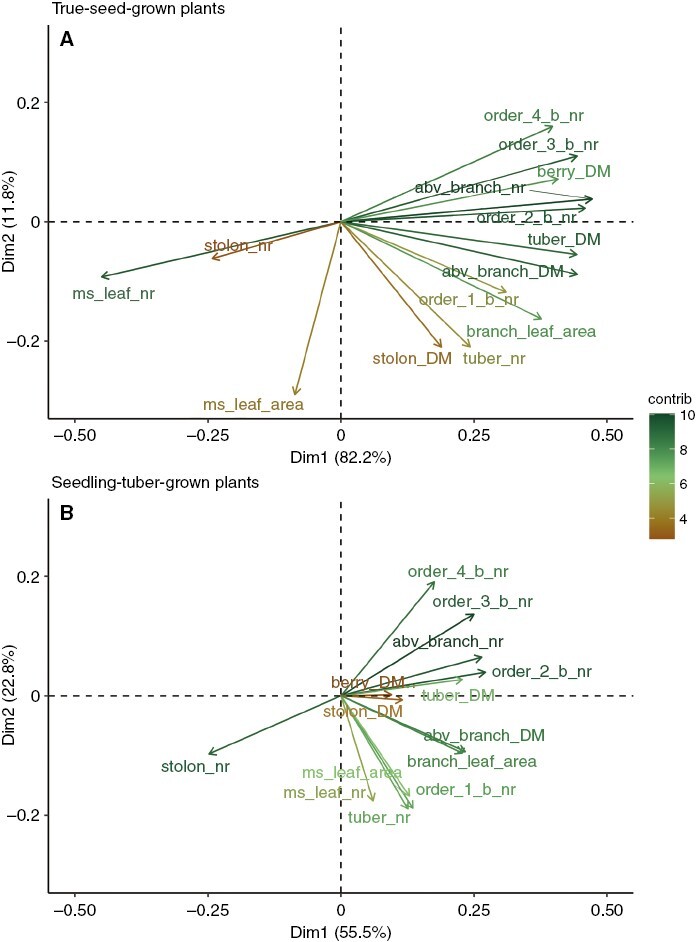
Principal component analysis of the associations between architectural traits for true-seed-grown plants (A) and seedling-tuber-grown plants (B). The longer the arrows, the greater the contribution of the variable to the principal components.

The different biomass allocation regimes indicated the different associations between above- and below-ground branches in true-seed-grown and seedling-tuber-grown plants, which can be seen in the correlation matrix of these traits for two propagule types (Supplementary Data Tables S4 and S5). In addition, we carried out a principal component analysis to present the associations between important architectural traits (Fig. 7; Supplementary Data Table S6).

In true-seed-grown plants, the number of main stem leaves exhibited a strongly negative correlation with almost all the above-ground branch-related traits; the fewer leaves that grew on the main stem, the more branches were produced (Fig. 7A). However, in seedling-tuber-grown plants, when the number of main stem leaves was larger, more order 1 branches developed, but higher-order branches or the total number of branches were not influenced (Fig. 7B). Moreover, the associations among all branching orders were stronger in true-seed-grown plants, and as indicated by the arrows, these traits were grouped together, whereas in seedling-tuber-grown plants the branches belonging to order 0 (main stem) and order 1 were closely correlated, but not with higher-order branches.

Linking above ground with below ground, the more branches that developed, especially order 1 and 2 branches, the more tubers were produced for both propagule types, suggesting that branch production above ground is consistent with branch production below ground. This relationship is stronger in true-seed-grown plants than in seedling-tuber-grown plants. Furthermore, there was almost no correlation between the number of main-stem leaves and the number of tubers in true-seed-grown plants, but this relationship was strongly positive in seedling-tuber-grown plants.

## DISCUSSION

Our study describes systematically the plant architectural development for potato plants with the same genetic background but grown from contrasting types of propagules: true seeds and seedling tubers. The architectural differences between the two propagule types are mainly attributed to the temporal–spatial distribution of lateral branches above and below ground on the main axis, but the sum of branch-related traits at the whole-plant level is not significantly different. These differences develop gradually as the development of branches enhances, which is also reflected in the divergences of biomass allocation patterns. Overall, the progressive differentiation of lateral branches, over time and space, in their number, size and branching order gives rise to a more branched and compact architecture in true-seed-grown plants compared with the less branched and taller seedling-tuber-grown plants. The different branching behaviours indicate that the functional contributions of branches to whole-plant growth and development differ between two propagule types.

### An important role of lateral branching in true-seed-grown plants

All the traits related to branches contribute to a larger proportion of the overall variance in true-seed-grown plants (Fig. 7A), indicating that lateral branches play a more significant role in their whole-plant architecture and plant function than in seedling-tuber-grown plants. This is reflected in the above-ground shoot branching indexes and below-ground tuber size distribution. Shoot branching indexes, expressed as the ratio of branch leaf area to plant leaf area and the ratio of branch stem length to plant stem length, are used to differentiate the photosynthetic and resource exploration functions of branches from the main axis (i.e. Smith *et al.*, 2014; Bruy *et al.*, 2018). In comparison to seedling-tuber-grown plants, the higher indexes in true-seed-grown plants (Supplementary Data Table S1) imply that a greater functional role is played by branches to the whole plant, such as their contribution to whole-plant photosynthesis and light interception. For example, because senescence of the main stem leaves occurs earlier in true-seed-grown plants than in seedling-tuber-grown plants, the continual production of branches is crucial for maintaining the whole-plant leaf area for light capture. Below ground, the tubers being produced are smaller in size, which requires a higher number of tubers to achieve a nearly equivalent yield in comparison to seedling-tuber-grown plants (Fig. 5; Table 3).

Moreover, in potato plants, assimilate partitioning shifts from shoots to tubers after tuberization (Van Heemst, 1986). The rate of this shift affects shoot growth: the slower the rate, the more gradually the shoot growth ceases; the gradually decreased assimilate availability in the shoot promotes sympodial branching, which is related to the enhanced development of flower primordia into mature flowers, favouring sexual reproduction (Almekinders and Struik, 1996). Similar phenomena are found in other species with sympodial growth within or beyond the Solanaceae family, such as tomato, tobacco and soybean (Tian *et al.*, 2010; Park *et al.*, 2014; Gaarslev *et al.*, 2021). These three studies reported that the rate of meristem transition from vegetative to reproductive growth has significant impacts on the whole-plant architecture, fecundity and productivity. Our results show that the duration of the shift to reach the same amount of tuber yield was longer in true-seed-grown plants than in seedling-tuber-grown plants (Supplementary Data Table S3). The slow shift in biomass allocation led to more investment in sexual reproduction, resulting in enhanced production of true seeds, formed in potato berries after flowering. This is in line with the observed higher berry biomass in true-seed-grown plants than in seedling-tuber-grown plants (Table 4; Fig. 6). Hence, it indicates that they might have a specific reproductive mode for clonal and sexual growth.

### Preferences for clonal and sexual reproduction differ between types of propagules

Our results show that larger tubers (organs for clonal reproduction) are produced in seedling-tuber-grown plants, and only small amounts of resources are allocated to production of true seeds (organs for sexual reproduction), indicating that clonal growth is more prioritized than sexual reproduction, in comparison to true-seed-grown plants. This is a strategy similar to many perennial plants for which clonal reproduction is more favoured over sexual propagation, because it is more competitive to expand an established population (Yang and Kim, 2016). The same authors also argued that sexual propagation can create new genetic variation to ensure survival, which can be the case in true-seed-grown plants. The more branched architecture above and below ground in true-seed-grown plants promotes the production of both true seeds and tubers, which implies that sexual and asexual reproduction are both favoured. This offers advantages for the dispersal and survival of such species, thus enhancing their resilience to environmental fluctuations. These characteristics are also present in many wild potato species, such as *Solanum oxycarpum* and *Solanum clarum*, which typically produce a bushy architecture, small tubers in large quantity and rely on sexual reproduction (Spooner *et al.*, 2004; Hardigan *et al.*, 2017).

### Distinct branching behaviours might result from the different intensity of apical dominance

When grown from true seeds, the development of branches does not appear to be constrained strictly by a specific quantity, location or defined time frame, indicative of an indeterminate growth habit. In contrast, there appears to be a threshold of branch production in seedling-tuber-grown plants, at each branching order and at specific node positions, which indicates determinate growth. Moreover, the different growth habits suggest that the strength and the release of apical dominance within the main axis might vary between types of propagules.

More specifically, a stronger and a cascading release of apical dominance might exist in seedling-tuber-grown plants, which exhibit a hierarchy of branching. First, the development of branches is contingent upon their branching order, whereby the growth of higher-order branches commences once the increase in the number of the low-order branches ceases (Supplementary Data Table S2). Second, depending on the node positions on the main stem, the number of above-ground branches, leaf area and the highest branching order appear to be part of a fixed pattern, which shows the positional preferential development of lateral axes on the parent axis (Figs 3 and 4). This sequential development of shoot branches and the positional effect have also been observed in potato plants grown from seed tubers (Almekinders and Struik, 1994) and in other Solanaceae species, such as tobacco (McDaniel and Hsu, 1976), in addition to Rosaceae species (Costes *et al.*, 2014). Third, the same phenomena can also be observed below ground in tuber size distribution (Fig. 5). A certain number of tubers stop bulking when a certain size is attained, resulting in an approximately normal size distribution, which has been also described by Struik *et al.* (1991) and suggests that a size hierarchy of tubers on the same stem might exist in tuber-grown plants.

Such sequential and positional development of branches, however, does not occur in true-seed-grown plants, indicating weak apical dominance. Similar quantities of above-ground branches and leaf area are produced across the majority of node positions, and the development of branches from any order can be observed during the same period (Figs 3 and 4). Nonetheless, tubers grow to a similar size. This is also in line with Struik *et al.* (1991), who suggest that different hierarchical mechanisms might underlie this phenomenon in plants grown from other types of propagules, such as true seeds or micro tubers.

Besides, at the molecular level, apical dominance-regulated branching control has been studied in potato plants (i.e. Hubbard *et al.*, 2002; González-Grandío *et al.*, 2013; Brewer, 2015; Nicolas *et al.*, 2015). For example, the *BRC1/TB1* gene and an alternative splicing in the *BRC1a* gene play important roles in inhibiting bud outgrowth in potato plants. Roumeliotis *et al.* (2012) proposed that the collaborative role of auxin and strigolactone in the control of the outgrowth of buds into stolons and/or tubers is similar to the regulation of shoot branching. It could be one of the reasons to explain the more branched architecture in true-seed-grown plants both above and below ground in the present study. In addition, Tao *et al.* (2010) reported that an enhanced cytokinin level was related to an increased number of tubers with reduced size, decreased main stem height and promoted lateral branching. These architectural traits agree with our observations in true-seed-grown plants, suggesting that a cytokinin-regulated apical dominance could also play a role in architectural differences between the two propagules. It is worth conducting further research on this genetic pathway and hormone signalling, which might provide more insights on the branching control/apical dominance in plants grown from different propagules with the same genotype. Moreover, our study provides a valid argument that types of propagules can alter architectural phenotypes, expressed as changes in branching behaviours. Given that the genes that regulate branching are rather conserved among many species, we propose that the type of propagule should be considered when studying branching control in plants.

### Concluding remarks

Our study quantified the differences in plant architectural development between two contrasting types of propagules, using the potato plant as an example. The more branched and compact architecture in true-seed-grown plants indicates a more significant functional role of branching, compared with that of seedling-tuber-grown plants. Considering a potato plant as a perennial herb, we surmise that a divergent branching behaviour in true-seed-grown plants could be a consequence of the existence of both clonal and sexual modes of reproduction. Moreover, the different branching patterns in two types of propagules might be regulated by the different intensity of apical dominance. A stronger and cascading release of apical dominance might exist in seedling-tuber-grown plants. We recommend that the type of propagule should be considered in future research to gain a better understanding of the physiological and molecular mechanisms in branching control.

## SUPPLEMENTARY DATA

Supplementary data are available at *Annals of Botany* online and consist of the following.

Figure S1: the distribution of the number of tubers and tuber fresh weight (in grams) in five tuber size classes at four developmental stages, for true-seed-grown and seedling-tuber-grown plants.

Table S1: whole-plant architectural traits per plant at four developmental stages for two propagule types.

Table S2: distribution of number of branches and leaf area at the whole-plant level, based on branching order and branch location on the main stem, in two propagule types at four developmental stages.

Table S3: biomass allocation (in grams) in different types of organs per plant in two propagule types at four developmental stages.

Table S4: correlation matrix among architectural traits and biomass allocated to different organs in true-seed-grown plants.

Table S5: correlation matrix among architectural traits and biomass allocated to different organs in seedling-tuber-grown plants.

Table S6: factor loadings for principal components 1 and 2, in true-seed-grown and seedling-tuber-grown plants.

mcad194_suppl_Supplementary_Material
