# Supplementary material for: Quantifying differences in plant architectural development between hybrid potato (Solanum tuberosum) plants grown from two types of propagules
Source: Ann Bot. 2023 Dec 14;133(2):365–78. doi: 10.1093/aob/mcad194 (PMC11005760; doi:10.1093/aob/mcad194)
Supplement: mcad194_suppl_Supplementary_Material [file mcad194_suppl_supplementary_material.docx]

| Table S1. Whole-plant architectural traits per plant (mean ± SD) at four developmental stages for two propagule types. Mean values from 8 plants. Different letters indicate significant differences between propagule types. Significant differences are in bold. | | | | | |
| --- | --- | --- | --- | --- | --- |
| **Plant traits** | **Propagule type** | **Week 3** | **Week 6** | **Week 9** | **Week 12** |
| Plant height (cm) | True seed | 21.8 ± 7.2 | 51.4 ± 2.5 | 66.0 ± 5.4 | **60.9 ± 5.1 a** |
|  | Seedling tuber | 22.3 ± 7.1 | 53.3 ± 5.3 | 69.4 ± 7.7 | **74.5 ± 8.8 b** |
| Main stem height (cm) | True seed | 19.1 ± 5.5 | **27.4 ± 4.3 a** | **30.9 ± 6.3 a** | **24.3 ± 3.9 a** |
|  | Seedling tuber | 20.0 ± 4.8 | **43.7 ± 6.1 b** | **46.1 ± 4.7 b** | **47.4 ± 4.5 b** |
| Branch length (cm) | True seed | 71.4 ± 27.6 | 286 ± 37.0 | 461 ± 91.7 | 675 ± 147 |
|  | Seedling tuber | 54.0 ± 24.0 | 312 ± 54.7 | 556 ± 139. | 594 ± 146 |
| Branch length: plant stem length | True seed | **78.2% ± 4.0% b** | **91% ± 1.8% b** | 93.5% ± 1.7% | **96.4% ± 0.8% b** |
|  | Seedling tuber | **71.5% ± 5.8% a** | **87.5% ± 2.3% a** | 92.0% ± 2.0% | **92.3% ± 1.9% a** |
| Number of branches aboveground | True seed | 4.75 ± 1.0 | 8.50 ± 3.6 | **20.6 ± 7.8 a** | 38.0 ± 11.1 |
|  | Seedling tuber | 4.38 ± 0.5 | 10.9 ± 2.2 | **32.0 ± 13.5 b** | 34.9 ± 13.2 |
| Number of leaves per plant | True seed | 44 ± 14 | 106 ± 5 | 187 ± 52 | 272 ± 55 |
|  | Seedling tuber | 43 ± 10 | 117 ± 16 | 227 ± 67 | 247 ± 67 |
| Green leaf area (cm^2^) | True seed | 1660 ± 390 | 4011 ± 235 | 5701 ± 628 | 5565 ± 783 |
|  | Seedling tuber | 1394 ± 336 | 4162 ± 293 | 5971 ± 429 | 5966 ± 543 |
| Branch leaf area: plant leaf area | True seed | 23.1% ± 6.8% | 51.7% ± 5.0% | 64.5% ± 2.9% | **72.1% ± 3.6% b** |
|  | Seedling tuber | 22.0% ± 9.4% | 52.2% ± 5.7% | 62.9% ± 5.1% | **63.7% ± 4.3% a** |
| Number of stolons | True seed | 13.3 ± 3.10 | 13.1 ± 3.31 | 13.1 ± 3.23 | 11.6 ± 2.62 |
|  | Seedling tuber | 10.6 ± 3.42 | 12.9 ± 2.42 | 11.2 ± 1.49 | 10.5 ± 1.85 |
| Number of tubers | True seed | 0.7 ± 1.7 | 25.0 ± 4.8 | **31.1 ± 10.2 b** | **31.4 ± 8.0 b** |
|  | Seedling tuber | 0.3 ± 0.7 | 22.2 ± 6.3 | **19.8 ± 8.5 a** | **24.6 ± 6.3 a** |
| Total number of branches (above-ground + below-ground branches) | True seed | 18.8 ± 3.65 | 46.6 ± 6.37 | 64.9 ± 14.6 | 81 ± 11.5 |
|  | Seedling tuber | 15.2 ± 3.62 | 46 ± 5.76 | 63.0 ± 16.9 | 70 ± 15.0 |

| Table S2. Distribution of number of branches and leaf area (mean ± SD) at whole-plant, based on branching order and branch location on the main stem, in two propagule types at four developmental stages. | | | | | |
| --- | --- | --- | --- | --- | --- |
| **Plant traits** | **Propagule type** | **Week 3** | **Week 6** | **Week 9** | **Week 12** |
| **Number of above-ground branches** |  |  |  |  |  |
| Whole plant | True seed | 4.75 ± 1.03 | 8.50 ± 3.59 | **20.6 ± 7.76 a** | 38.0 ± 11.1 |
|  | Seedling tuber | 4.38 ± 0.52 | 10.9 ± 2.17 | **32.0 ± 13.5 b** | 34.9 ± 13.2 |
| Based on location on main stem^1^ |  |  |  |  |  |
| *Basal branch* | True seed | 4.75 ± 1.03 | 5.50 ± 2.27 | **13.1 ± 4.52 a** | 24 ± 7.67 |
|  | Seedling tuber | 4.38 ± 0.52 | 8.75 ± 1.83 | **23.2 ± 9.38 b** | 25.5 ± 9.78 |
| *Apical branch* | True seed | 0 | 3.00 ± 1.60 | 7.50 ±3.89 | **14.0 ± 4.60 b** |
|  | Seedling tuber | 0 | 2.12 ± 0.35 | 8.75 ± 4.43 | **9.38 ± 3.66 a** |
|  |  |  |  |  |  |
| Based on branching order^2^ |  |  |  |  |  |
| *Order1 branches* | True seed | 4.75 ± 1.04 | 7.88 ± 3.23 | 9.75 ± 1.04 | 10.38 ± 1.06 |
|  | Seedling tuber | 4.38 ± 0.52 | 10.0 ± 0.76 | 10.1 ± 1.73 | 9.88 ± 0.64 |
| *Order 2 branches* | True seed | 0 | 0.63 ± 0.92 | **8.63 ± 4.63 a** | **16.5 ± 4.69 b** |
|  | Seedling tuber | 0 | 0.88 ± 2.48 | **13.4 ± 4.87 b** | **12.6 ± 2.98 a** |
| *Order 3 branches* | True seed | 0 | 0 | **2.25 ± 2.60 a** | 9.25 ± 5.80 |
|  | Seedling tuber | 0 | 0 | **7.63 ± 6.82 b** | 10.13 ± 7.74 |
| *Order 4 branches* | True seed | 0 | 0 | 0 | 1.88 ± 1.73 |
|  | Seedling tuber | 0 | 0 | 0.88 ± 1.81 | 2.25 ± 2.77 |
|  |  |  |  |  |  |
| **Leaf area (cm^2^)** |  |  |  |  |  |
| Whole plant | True seed | 1660 ± 390 | 4011 ± 235 | 5701 ± 628 | 5565 ± 783 |
|  | Seedling tuber | 1394 ± 336 | 4162 ± 293 | 5971 ± 429 | 5966 ± 543 |
| Based on location on main stem^1^ |  |  |  |  |  |
| *Basal leaves* | True seed | 1406 ± 286 | **2772 ± 231 a** | **3865 ± 447 a** | **3652 ± 645 a** |
|  | Seedling tuber | 1335 ± 306 | **3449 ± 286 b** | **4596 ± 256 b** | **4650 ± 506 b** |
| *Apical leaves* | True seed | 254 ± 159 | **1239 ± 145 b** | **1836 ± 349 b** | **1913 ± 204 b** |
|  | Seedling tuber | 59.6 ± 50.6 | **713 ± 119 a** | **1375 ± 363 a** | **1316 ± 210 a** |
|  |  |  |  |  |  |
| Based on branching order^2^ |  |  |  |  |  |
| *Main stem (Order 0)* | True seed | 1260 ± 245 | 1943 ± 252 | 2019 ± 229 | **1557 ± 315 a** |
|  | Seedling tuber | 1063 ± 151 | 1987 ± 263 | 2213 ± 346 | **2165 ± 325 b** |
| *Order1 branches* | True seed | 399 ± 185 | 2063 ± 188 | 3179 ± 344 | 3070 ± 382 |
|  | Seedling tuber | 331 ± 224 | 2158 ± 272 | 3106 ± 311 | 3034 ± 466 |
|  |  |  |  |  |  |
| *Order 2 branches* | True seed | 0 | 4.62 ± 13.1 | 465 ± 226 | 775 ± 190 |
|  | Seedling tuber | 0 | 17.7 ± 50.0 | 569 ± 243 | 651 ± 172 |
| *Order 3 branches* | True seed | 0 | 0 | 38.4 ± 43.2 | 148 ± 88.4 |
|  | Seedling tuber | 0 | 0 | 79.2 ± 84.1 | 108 ± 92.1 |
| *Order 4 branches* | True seed | 0 | 0 | 0 | 14.0 ± 13.6 |
|  | Seedling tuber | 0 | 0 | 3.56 ± 7.19 | 8.22 ± 15.6 |
| ^1^ distribution of number of branches or leaf area based on branching order  ^2^ distribution of number of branches or leaf area based on branch locations on the main stem | | | | | |

Fig. S1. The distribution of number of tubers (Panel A—H) and tuber fresh weight (g) (Panel I—P) in 5 tuber size classes at four developmental stages, for true-seed-grown and seedling-tuber-grown plants (mean values of 8 plants).


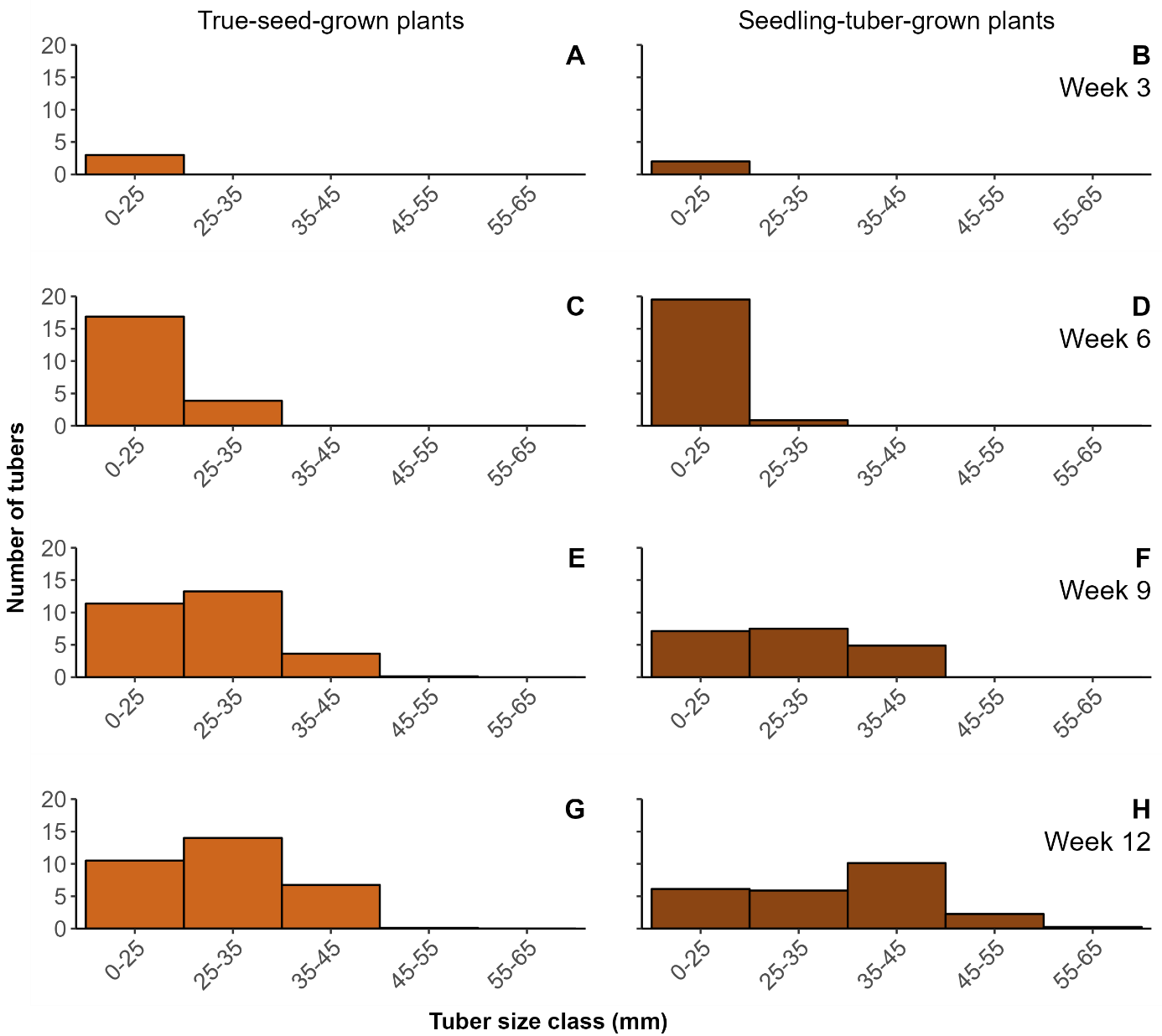


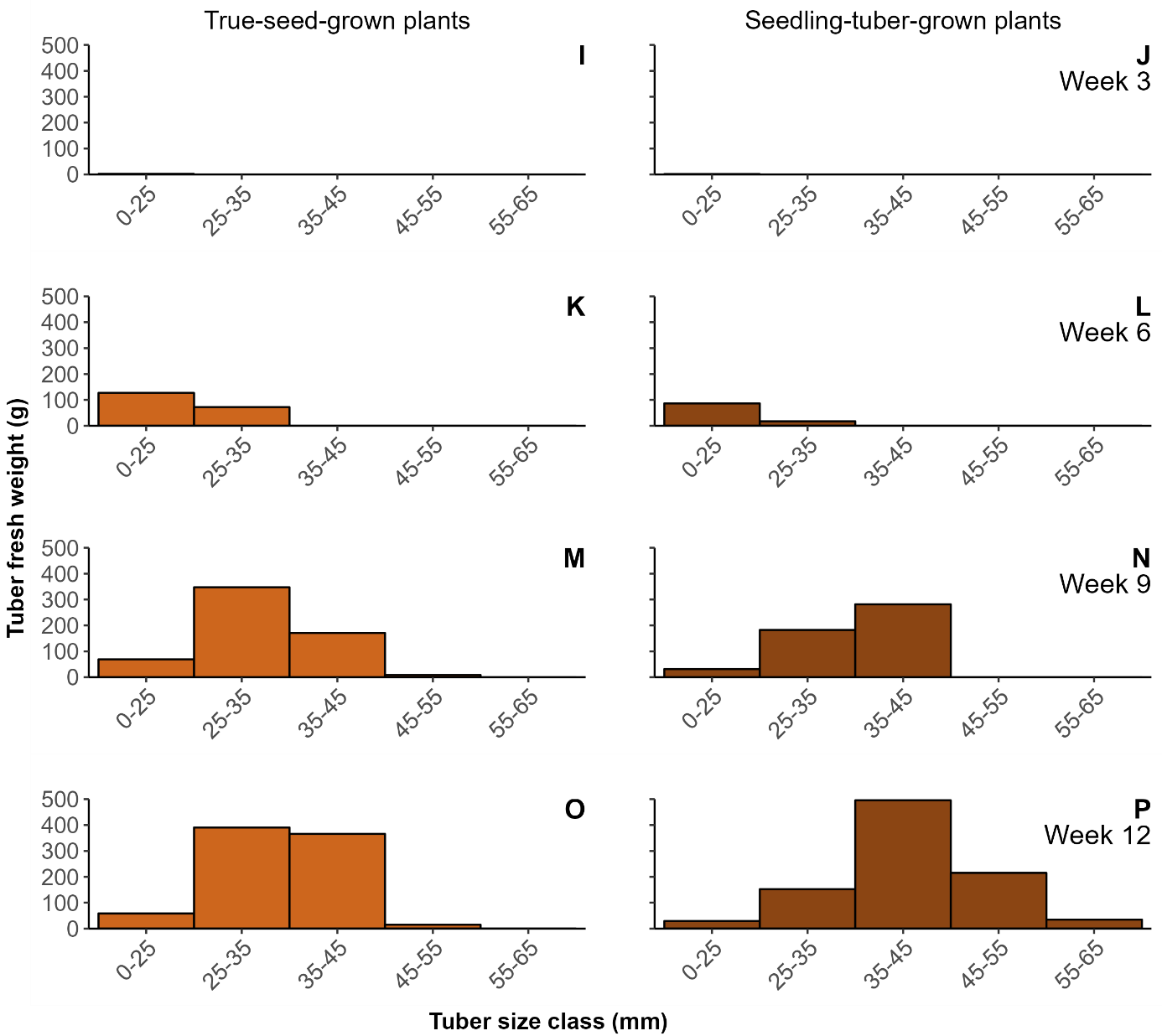


| Table S3. Biomass allocation (g) in different types of organs per plant (n = 8) in two propagule types at four developmental stages. | | | | | |
| --- | --- | --- | --- | --- | --- |
| **Plant traits** | **Propagule type** | **Week 3** | **Week 6** | **Week 9** | **Week 12** |
| **Total biomass per plant** | True seed | 13.5 ± 5.18 | 69.8 ± 6.25 | 177 ± 9.69 | 285 ± 22.8 |
|  | Seedling tuber | 11.0 ± 4.36 | 64.7 ± 4.46 | 166 ± 17.6 | 294 ± 21.5 |
|  |  |  |  |  |  |
| **Above-ground part** | True seed | 10.5 ± 4.00 | **27.8 ± 2.87 a** | **47.3 ± 8.28 a** | **63.5 ± 11.1 b** |
|  | Seedling tuber | 8.71 ± 3.18 | **37.6 ± 3.71 b** | **57.4 ± 4.60 b** | **56.3 ± 9.10 a** |
| Main stem (stem + leaves) | True seed | 7.95 ± 2.70 | **14.0 ± 2.27 a** | **17.0 ± 1.56 a** | **15.4 ± 2.36 a** |
|  | Seedling tuber | 6.71 ± 1.70 | **19.0 ± 2.44 b** | **23.6 ± 2.70 b** | **20.9 ± 1.18 b** |
| Berries | True seed | 0 | 0 | 4.38 ± 7.44 | 13.3 ± 8.99 b |
|  | Seedling tuber | 0 | 0 | 1.92 ± 2.74 | 5.50 ± 4.49 a |
| Total branch (stem + leaves) | True seed | 2.54 ± 1.40 | **13.9 ± 1.60 a** | **25.9 ± 4.06 a** | **34.8 ± 5.74 b** |
|  | Seedling tuber | 2.00 ± 1.57 | **18.6 ± 2.57 b** | **31.9 ± 4.10 b** | **29.9 ± 5.60 a** |
| Based on branching order^1^ |  |  |  |  |  |
| *Order 1 branches* | True seed | 2.54 ± 1.40 | **13.8 ± 1.56 a** | **22.2 ± 2.09 a** | 26.2 ± 3.20 |
|  | Seedling tuber | 2.00 ± 1.57 | **18.4 ± 2.45 b** | **25.3 ± 3.27 b** | 23.8 ± 4.73 |
| *Order 2 branches* | True seed | 0 | 0.049 ± 0.138 | **3.33 ± 1.81 a** | **6.57 ± 1.71 b** |
|  | Seedling tuber | 0 | 0.177 ± 0.501 | **5.00 ± 2.07 b** | **4.79 ± 1.47 a** |
| *Order 3 branches* | True seed | 0 | 0 | **0.419 0.477 a** | 1.77 1.05 |
|  | Seedling tuber | 0 | 0 | **1.50 ± 1.46 b** | 1.18 ± 1.05 |
| *Order 4 branches* | True seed | 0 | 0 | 0 | 0.227 ± 0.205 |
|  | Seedling tuber | 0 | 0 | 0.084 ± 0.164 | 0.139 ± 0.213 |
|  |  |  |  |  |  |
| **Below-ground part** | True seed | 2.98 ± 1.29 | **42.0 ± 6.48 b** | **130 ± 14.9 b** | **219 ± 26.0 a** |
|  | Seedling tuber | 2.32 ± 1.18 | **26.9 ± 3.28 a** | **109 ± 19.8 a** | **237 ± 21.4 b** |
| Tubers | True seed | 0.074 ± 0.194 | **33.3 ± 8.23 b** | **119.0 ± 15.7 b** | 210.0 ± 26.7 |
|  | Seedling tuber | 0.028 ± 0.08 | **16.2 ± 4.48 a** | **94.8 ± 20.1 a** | 224.0 ± 28.9 |
| Stolons | True seed | 0.579 ± 0.311 | 3.93 ± 1.38 | 5.24 ± 1.46 | 4.26 ± 2.54 |
|  | Seedling tuber | 0.336 ± 0.34 | 4.15 ± 1.83 | 5.78 ± 2.93 | 6.68 ± 6.81 |
| Roots | True seed | 2.32 ± 0.974 | **4.75 ± 0.971 a** | **5.78 ± 1.24 a** | 5.56 ± 1.88 |
|  | Seedling tuber | 1.96 ± 0.898 | **6.57 ± 1.97 b** | **8.34 ± 1.23 b** | 6.49 ± 1.64 |
| ^1^ Total branch biomass distributed to different branches based on branching order | | | | | |

Table S4. Correlation matrix among architectural traits and biomass allocated to different organs in true-seed-grown plant.

| abv_branch_DM | 0.91*** | 1 |  |  |  |  |  |  |  |  |  |  |  |  |
| --- | --- | --- | --- | --- | --- | --- | --- | --- | --- | --- | --- | --- | --- | --- |
| ms_leaf_nr | 0.65*** | 0.62*** | 1 |  |  |  |  |  |  |  |  |  |  |  |
| order_1_b_nr | 0.72*** | 0.76*** | -0.3 | 1 |  |  |  |  |  |  |  |  |  |  |
| order_2_b_nr | 0.97*** | 0.90*** | -0.66*** | 0.66*** | 1 |  |  |  |  |  |  |  |  |  |
| order_3_b_nr | 0.93*** | 0.77*** | -0.61*** | 0.48** | 0.86*** | 1 |  |  |  |  |  |  |  |  |
| order_4_b_nr | 0.76*** | 0.59*** | -0.62*** | 0.34 | 0.63*** | 0.87*** | 1 |  |  |  |  |  |  |  |
| ms_leaf_area | 0.05 | 0.32 | 0.32 | 0.28 | 0.03 | -0.03 | -0.18 | 1 |  |  |  |  |  |  |
| branch_leaf_area | 0.79*** | 0.96*** | -0.49** | 0.78*** | 0.79*** | 0.62*** | 0.40* | 0.48** | 1 |  |  |  |  |  |
| stolon_nr | -0.24 | -0.22 | 0.14 | -0.19 | -0.17 | -0.3 | -0.27 | 0.13 | -0.16 | 1 |  |  |  |  |
| tuber_nr | 0.53** | 0.76*** | -0.33 | 0.63*** | 0.51** | 0.38* | 0.33 | 0.59*** | 0.83*** | 0.03 | 1 |  |  |  |
| stolon_DM | 0.47** | 0.67*** | -0.24 | 0.52** | 0.46** | 0.35 | 0.23 | 0.63*** | 0.71*** | 0.02 | 0.73*** | 1 |  |  |
| tuber_DM | 0.84*** | 0.91*** | -0.68*** | 0.69*** | 0.86*** | 0.68*** | 0.53** | 0.16 | 0.89*** | -0.16 | 0.70*** | 0.47** | 1 |  |
| berry_DM | 0.61*** | 0.57*** | -0.68*** | 0.36* | 0.58*** | 0.60*** | 0.59*** | -0.08 | 0.48** | -0.45** | 0.37* | 0.39* | 0.60*** | 1 |
|  | abv_branch_nr | abv_branch_DM | ms_leaf_nr | order_1_b_nr | order_2_b_nr | order_3_b_nr | order_4_b_nr | ms_leaf_area | branch_leaf_area | stolon_nr | tuber_nr | stolon_DM | tuber_DM | berry_DM |

Table S5. Correlation matrix among architectural traits and biomass allocated to different organs in seedling-tuber-grown plants.

| abv_branch_nr | 1 |  |  |  |  |  |  |  |  |  |  |  |  |  |
| --- | --- | --- | --- | --- | --- | --- | --- | --- | --- | --- | --- | --- | --- | --- |
| abv_branch_DM | 0.81*** | 1 |  |  |  |  |  |  |  |  |  |  |  |  |
| ms_leaf_nr | 0.48** | 0.53** | 1 |  |  |  |  |  |  |  |  |  |  |  |
| order_1_b_nr | 0.65*** | 0.83*** | 0.72*** | 1 |  |  |  |  |  |  |  |  |  |  |
| order_2_b_nr | 0.94*** | 0.82*** | 0.41* | 0.59*** | 1 |  |  |  |  |  |  |  |  |  |
| order_3_b_nr | 0.95*** | 0.63*** | 0.37* | 0.47** | 0.83*** | 1 |  |  |  |  |  |  |  |  |
| order_4_b_nr | 0.75*** | 0.43* | 0.23 | 0.27 | 0.55** | 0.84*** | 1 |  |  |  |  |  |  |  |
| ms_leaf_area | 0.59*** | 0.77*** | 0.61*** | 0.83*** | 0.57*** | 0.43* | 0.24 | 1 |  |  |  |  |  |  |
| branch_leaf_area | 0.80*** | 0.98*** | 0.55** | 0.80*** | 0.83*** | 0.61*** | 0.42* | 0.79*** | 1 |  |  |  |  |  |
| stolon_nr | -0.1 | -0.03 | 0.23 | 0.2 | -0.13 | -0.15 | -0.11 | 0.14 | -0.01 | 1 |  |  |  |  |
| tuber_nr | 0.52** | 0.75*** | 0.63*** | 0.80*** | 0.48** | 0.36* | 0.22 | 0.76*** | 0.79*** | 0.11 | 1 |  |  |  |
| stolon_DM | 0.52** | 0.60*** | 0.32 | 0.49** | 0.42* | 0.47** | 0.51** | 0.56*** | 0.55** | -0.01 | 0.53** | 1 |  |  |
| tuber_DM | 0.71*** | 0.68*** | 0.24 | 0.49** | 0.75*** | 0.61*** | 0.44* | 0.56*** | 0.77*** | -0.16 | 0.55** | 0.34 | 1 |  |
| berry_DM | 0.28 | 0.44* | -0.08 | 0.21 | 0.35 | 0.18 | 0.13 | 0.51** | 0.50** | -0.08 | 0.37* | 0.44* | 0.66*** | 1 |
|  | abv_branch_nr | abv_branch_DM | ms_leaf_nr | order_1_b_nr | order_2_b_nr | order_3_b_nr | order_4_b_nr | ms_leaf_area | branch_leaf_area | stolon_nr | tuber_nr | stolon_DM | tuber_DM | berry_DM |

Table S6. Factor loadings for Principal components 1 and 2, in true-seed-grown and seedling-tuber-grown plants.

| **Factor loadings** | | | | | |
| --- | --- | --- | --- | --- | --- |
| Variables | **True-seed-grown plants** | |  | **Seedling-tuber-grown plants** | |
|  | Comp.1 | Comp.2 |  | Comp.1 | Comp.2 |
| aboveground_branch_nr | 0.338086 | 0.071886 |  | 0.362215 | 0.138158 |
| aboveground_branch_DM | 0.318039 | -0.16664 |  | 0.320064 | -0.199640 |
| ms_leaf_nr | -0.32217 | -0.17493 |  | 0.082716 | -0.375230 |
| order_1_b_nr | 0.222107 | -0.22326 |  | 0.185002 | -0.401870 |
| order_2_b_nr | 0.328798 | 0.043101 |  | 0.371482 | 0.084071 |
| order_3_b_nr | 0.317767 | 0.207899 |  | 0.341401 | 0.291875 |
| order_4_b_nr | 0.284880 | 0.302209 |  | 0.240297 | 0.407685 |
| ms_leaf_area | -0.061940 | -0.546960 |  | 0.175937 | -0.358030 |
| branch_leaf_area | 0.269724 | -0.307240 |  | 0.312127 | -0.204760 |
| berry_DM | 0.292277 | 0.135177 |  | 0.130002 | 0.004437 |
| stolon_nr | -0.173440 | -0.116700 |  | -0.339750 | -0.207703 |
| tuber_nr | 0.173736 | -0.39593 |  | 0.172322 | -0.404200 |
| stolon_DM | 0.134950 | -0.39657 |  | 0.158245 | -0.015210 |
| tuber_DM | 0.317323 | -0.10354 |  | 0.312324 | 0.058292 |
